# Microsatellite Development for an Endangered Bream *Megalobrama pellegrini* (Teleostei, Cyprinidae) Using 454 Sequencing

**DOI:** 10.3390/ijms13033009

**Published:** 2012-03-06

**Authors:** Jinjin Wang, Xiaomu Yu, Kai Zhao, Yaoguang Zhang, Jingou Tong, Zuogang Peng

**Affiliations:** 1 The Key Laboratory of Freshwater Fish Reproduction and Development (Ministry of Education), Key Laboratory of Aquatic Science of Chongqing, School of Life Science, Southwest University, Beibei, Chongqing 400715, China; E-Mails: wangjin87@126.com (J.W.); zhangyg@swu.edu.cn (Y.Z.); 2 The State Key Laboratory of Freshwater Ecology and Biotechnology, Institute of Hydrobiology, The Chinese Academy of Sciences, Wuhan 430072, China; E-Mail: xmyu@ihb.ac.cn; 3 The Key Laboratory of Adaptation and Evolution of Plateau Biota (AEPB), Northwest Plateau Institute of Biology, The Chinese Academy of Sciences, Xining 810001, China; E-Mail: zhaokai@nwipb.cas.cn

**Keywords:** high-throughput microsatellite isolation, next-generation sequencing, breams, cross-amplification

## Abstract

*Megalobrama pellegrini* is an endemic fish species found in the upper Yangtze River basin in China. This species has become endangered due to the construction of the Three Gorges Dam and overfishing. However, the available genetic data for this species is limited. Here, we developed 26 polymorphic microsatellite markers from the *M. pellegrini* genome using next-generation sequencing techniques. A total of 257,497 raw reads were obtained from a quarter-plate run on 454 GS-FLX titanium platforms and 49,811 unique sequences were generated with an average length of 404 bp; 24,522 (49.2%) sequences contained microsatellite repeats. Of the 53 loci screened, 33 were amplified successfully and 26 were polymorphic. The genetic diversity in *M. pellegrini* was moderate, with an average of 3.08 alleles per locus, and the mean observed and expected heterozygosity were 0.47 and 0.51, respectively. In addition, we tested cross-species amplification for all 33 loci in four additional breams: *M. amblycephala*, *M. skolkovii*, *M. terminalis*, and *Sinibrama wui*. The cross-species amplification showed a significant high level of transferability (79%–97%), which might be due to their dramatically close genetic relationships. The polymorphic microsatellites developed in the current study will not only contribute to further conservation genetic studies and parentage analyses of this endangered species, but also facilitate future work on the other closely related species.

## 1. Introduction

*Megalobrama pellegrini* is a cyprinid fish species belonging to Cultrinae (Cypriniformes), which is endemic to China. It is distributed in the main streams and tributaries along the upper reaches of the Yangtze River [1,2] in the Sichuan basin. Recently, this species has become endangered as a consequence of a sharp decrease in the population size due to overfishing and/or the loss of habitats following completion of the Three Gorges Dam and several other dams along the upper Yangtze River [3–6], as *M. pellegrini* must inhabit flowing waters, especially in the breeding season [5]. Field surveys revealed that only one wild population of *M. pellegrini* had been found in Longxi River, one of the tributaries of the upper Yangtze River [5]. However, the studies on *M. pellegrini* are limited, and most studies have been restricted to artificial propagation, population ecology, and molecular endocrinology [5–10]. Regarding genetic studies, only Liu and Wang [11] studied the genetic structure of this species based on allozyme markers and indicated that *M. pellegrini* has a relatively high level of genetic variation, especially compared with the congeneric species Wuchang bream, *M. amblycephala*. However, no other molecular markers are available for the study of population and conservation genetics in this species. Furthermore, the validity of species in *Megalobrama* has long been a controversial subject because of the extraordinary similarities in morphological traits of the members of this genus [12,13]. *M. pellegrin* had been treated as being synonymous with *M. terminalis*, but recently several studies have recognized this to be a valid species, and based on morphological data, have clarified that the *Megalobrama* genus includes four valid species: *M. amblycephala*, *M. pellegrini*, *M. skolkoii*, and *M. terminalis* [1,12]. In short, the development of molecular markers to reveal population and conservation genetic studies, parentage analyses or a genetic map for this endangered species is required.

Microsatellites, or simple sequence repeats (SSRs), have been used widely since the late eighties for applications such as parentage analyses, population genetic structure and conservation genetics because of their high level of polymorphism, relatively small size and rapid analysis protocol [14–17]. However, the development of microsatellite loci using traditional methods is not only costly and time-consuming [18] but also limited by the difficulties of *de novo* development in species without any genomic information. Recently, the emergence of next-generation sequencing technologies has rapidly improved SSR development. Among next-generation sequencing technologies, the 454 GS-FLX technology (Roche Applied Science) has created new opportunities and made high-throughput microsatellite development cheaper and faster [19–28]. The first reports of this promising application were published in 2009 [19–21]. In addition, compared with single-nucleotide polymorphisms (SNPs), which have been increasingly used since the late nineties, SSRs offer high allelic diversity and the relative ease of transfer between closely related species [29]. Hence, SSRs could remain relevant genetic markers, at least for some specific applications.

Here, we present the development of 26 polymorphic species-specific microsatellite loci for the endangered species *M. pellegrini* based on the 454 sequencing technology. Subsequently, we tested all 33 microsatellite loci (including 26 poly- and 7 monomorphic microsatellite loci in *M. pellegrini*) in four other related species: *M. amblycephala*, *M. skolkoii*, and *M. terminalis* from the same genus, and one species, *Sinibrama wui*, from a closely related genus, none of which had previously published microsatellite primers, except for *M. amblycephala* [30]. The microsatellite markers described herein offer important genetic resources for the assessment, understanding and conservation of the endangered species *M. pellegrini*, and facilitate future work on the other related species.

## 2. Results and Discussion

### 2.1. Results

#### 2.1.1. 454 Sequencing Results

The raw sequence data from the 1/4 run of 454 sequencing were 90.2 Mbp containing 257,497 reads/sequences with an average length of 367 bp (maximum: 644 bp, minimum: 21 bp). The raw sequences represented large numbers of individual sequence reads, which could be assembled into contigs. A total of 208,525 reads were assembled into 839 contigs with an average length of 847 bp (maximum: 9,191 bp, minimum: 500 bp), leaving 48,972 singletons. The mean length of these 49,811 sequences (839 contigs plus 48,972 singletons) was 404 bp, which was slightly longer than that of the raw sequences. Of the 49,811 unique sequences, 24,522 (49.2%) sequences contained SSRs, and 14,987 (30.1%) sequences could be used for SSR primer design.

#### 2.1.2. Microsatellite Development

We selected 53 microsatellite loci (9 di-, 16 tri-, 25 tetra-nucleotide, and 3 compound repeats) from the contigs and singletons that were longer than 400 bp for subsequent polymorphism screening in *M. pellegrini*. Of the 53 microsatellite loci, 33 (62.3%) loci were amplified successfully (including 5 di-, 9 tri-, and 19 tetra-nucleotide repeats). Subsequently, further screening revealed that 26 (78.8%) loci were polymorphic in 8 *M. pellegrini* samples. The statistical results for these 26 polymorphic loci in a natural population of *M. pellegrini* from Longxi River are presented in Table 1. The number of alleles (N_A_) per locus ranged from 2 to 5 (mean 3.08 ± 1.02), and the observed (H_O_) and expected (H_E_) heterozygosity ranged from 0.07 to 0.80 (mean 0.47 ± 0.23) and 0.22 to 0.72 (mean 0.51 ± 0.13), respectively. The polymorphic information content (PIC) ranged from 0.20 to 0.67 (mean 0.42), with 8 (MP6/15/16/17/32/40/45/48) of these 26 loci revealing moderately high information content (0.51–0.67). Overall, the microsatellite markers isolated here showed a moderate level of polymorphism.

Deviation from Hardy-Weinberg equilibrium (HWE) was evident at 7 loci (MP6/9/11/26/34/41/46), and evidence of a null allele was also observed at 4 of these loci (MP6/26/41/46), with a 95% confidence interval. All loci observed in *M. pellegrini* were in linkage equilibrium, except for MP26 with MP30 (*P* < 0.05) (Table 1). All of the 26 polymorphic loci are accessible from GenBank with the accession No. JQ087470 JQ087495.

#### 2.1.3. Cross-species Amplification

**Figure 1:**
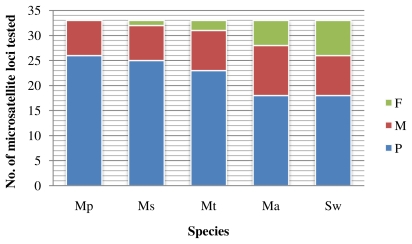
The result of tests for polymorphism and amplification across the five related species. Thirty-three microsatellite loci were screened for polymorphism. ‘P’ polymorphic, ‘M’ monomorphic, ‘F’ failed to amplify or multiple non-specific amplification. Species abbreviations: *M. pellegrini* ‘Mp’, *M. skolkoii* ‘Ms’, *M. terminalis* ‘Mt’, *M. amblycephala* ‘Ma’, and *Sinibrama wui* ‘Sw’.

The 33 microsatellite loci amplified successfully in *M. pellegrini* were further screened for cross-amplification in four other related species, *M. amblycephala*, *M. skolkoii*, *M. terminalis*, and *S. wui*. High success rates of cross-amplification were obtained: 97.0% (32/33) for *M. skolkoii*, 94.0% (31/33) for *M. terminalis*, 84.9% (28/33) for *M. amblycephala*, and 78.8% (26/33) for *S. wui* from a different genus (Figure 1). We found evidence for multiple alleles in 78.1% (25/32) for *M. skolkoii*, 74.2% (23/31) for *M. terminalis*, 64.3% (18/28) for *M. amblycephala*, and 69.2% (18/26) for *S. wui* (Figure 1).

### 2.2. Discussion

#### 2.2.1. 454 Sequencing Results

Longer reads may increase the likelihood of detecting microsatellite loci with more repeats, which are expected to be more polymorphic [27,31]. We assembled the raw sequences/reads to contigs to increase the sequences length and eliminate the repetitive sequences. Approximately 81.0% (208,525) of the 257,497 raw sequences were assembled into 839 contigs, which eliminated the repetitive sequences to a large extent. The average length of the contigs and singletons was slightly longer than that of the raw sequences (404 bp *vs.* 367 bp), which was long enough for the identification of SSRs and primer design [29]. Furthermore, the vast numbers of remaining sequences that did not contain microsatellite repeats would be useful for other purposes, such as molecular markers development for phylogenomic or ecological genomic studies.

#### 2.2.2. The Microsatellite Development from *M. pellegrini*

The development of polymorphic microsatellite using 454 sequencing technology has been an outstanding new and increasingly universal method, which can develop more polymorphic markers with lower costs and less time-consuming than traditional methods [29]. In the present study, we have successfully developed 26 polymorphic microsatellite loci for an endangered fish species. There were 7 loci that deviated from HWE significantly, which was most likely due to the presence of null alleles, as occurred in four of those markers. Other possible causes for the observed deviation could be homozygote and/or heterozygote excess (e.g., MP6, MP9, MP11, MP34, and MP46). Eight of the 26 polymorphic loci were assessed to contain moderately high (PIC > 0.50) polymorphism degree (Table 1), which could be used in further resolution of the population structure and other conservation genetic studies [32]. In addition, the development of reliable microsatellite markers with moderately high PIC for *M. pellegrini* is the first step in introducing marker-assisted selection for directed breeding programs, which would be useful for parentage assignment in the selected *M. pellegrini*.

The genetic diversity of *M. pellegrini* based on the 26 polymorphic microsatellite loci in this study was moderate (mean N_A_ = 3.08, H_O_ = 0.47, H_E_ = 0.51). Liu and Wang [11] also evaluated the genetic diversity of *M. pellegrini* using allozymes, and revealed that the mean heterozygosity was only 0.091, which was much lower than that from our study. This disparity was also observed in many other studies [33–36], which was likely caused by the different resolving power of different markers in the assessment of genetic diversity. The genetic diversity of *M. pellegrini* was similar to that of *M. amblycephala* (mean N_A_ = 2.9, H_O_ = 0.60, H_E_ = 0.55) [30]. In addition, the microsatellite diversity in *M. pellegrini* was slightly lower than those from other endangered fishes endemic to the upper Yangtze River basin, such as largemouth bronze gudgeon (*Coreius guichenoti*, mean N_A_ = 5.2, H_O_ = 0.42, H_E_ = 0.63) [37], Chinese rare minnow (*Gobiocypris rarus*, mean N_A_ = 4.4, H_O_ = 0.51, H_E_ = 0.65) [38], and rock carp (*Procypris rabaudi*, mean N_A_ = 6.9, H_O_ = 0.71, H_E_ = 0.77) [39].

*M. pellegrini* inhabits flowing waters and has strict habitat requirements, especially in the breeding season [5]. However, the Three Gorges Dam, constructed from 1994 and completed in 2006, has critically damaged the habitat by drastic changes in the environments; for example, the flow regimes were altered from free-flowing to stagnant, resulting in a substantial decline of biodiversity [3]. Furthermore, the increased human activity, particularly overfishing, is also a crucial factor for the population decline of *M. pellegrini* [40]. All of these factors have contributed to the sharp population decline of *M. pellegrini* in recent years [4,6]. Accordingly, the observed moderate genetic diversity in *M. pellegrini* (mean N_A_ = 3.08, H_O_ = 0.47, H_E_ = 0.51) might be caused by the severe decline of the population size in recent decades, which would be more severe if the wild population of this species continuously decreased. Hence, there is an urgent need to create effective management strategies for the conservation of wild populations of *M. pellegrini*. Artificial propagation and supplementation might be efficient approaches for the population recovery and preservation of the genetic diversity of this endemic species [3,40].

#### 2.2.3. High Level of Cross-species Amplification

The amplification success and cross-species transferability of the markers in this study were high (Table 1 and Figure 1), which might be due to the close genetic relationships among the tested species. The validity of the species in *Megalobrama* had been controversial for a long time; however, several studies [1,12,13] have recently clarified that there are four valid species in *Megalobrama: M. amblycephala*, *M. pellegrini*, *M. skolkoii*, and *M. terminalis*. Cai *et al*. [13] compared the differences of the morphological traits among the four species in *Megalobrama* and revealed that some of their traits were partly similar. Furthermore, the pairwise genetic distances among the four species are unbelievably low based on the complete mitochondrial genome dataset (Wang *et al.* unpublished data). Both comparisons from the morphological and molecular datasets suggest that the fishes in *Megalobrama* have close genetic relationships and might have diverged from their common ancestor relatively recently. Furthermore, the determination of the high cross-species transferability and levels of polymorphism of microsatellite loci from all breams herein will be worthwhile for population genetics studies without the need for expensive and time-consuming *de novo* microsatellite development.

## 3. Experimental Section

### 3.1. Sample Collection and 454 Sequencing

*M. pellegrini* samples were collected from the Longxi River, a tributary of the upper Yangtze River in Sichuan Province. Species identification was verified upon examination of morphological distinctions [1]. Fresh muscle tissues stored in 95% ethanol were used for the extraction of gDNA using the DNeasy Blood and Tissue kit (Qiagen). The quality and quantity of the gDNA were determined using an AstraGene Life Sciences Spectrophotometer (Astranet Systems Ltd, Newton, Cambridge, UK). The gDNA from a single sample was subjected to sequencing on a 1/4 plate in a 454 Life Sciences Genome Sequencer FLX Titanium instrument (Roche).

### 3.2. Microsatellite Discovery and Primer Screening

The resulting raw sequences for *M. pellegrini* were assembled into contigs using the Newbler 2.3 software. MSATCOMMANDER version 0.8.2 [41] was used to screen microsatellites with the default parameters (the minimum repeats were 10, 6, 4, 4, 4, and 4 for mono-, di-, tri-, tetra-, penta-, and hexa-nucleotide, respectively). A PERL script was performed to select sequences longer than 400 bp from among the contigs and singletons, which were searched for SSRs and further primer screening. Finally, the microsatellite loci used for the polymorphism screening were selected with additional constraints (minimum repeats of 14, 10, and 8 for the perfect di-, tri-, and tetra-nucleotide microsatellites, respectively) to increase the probability of polymorphism. The primers were designed using the Primer Premier software version 5 (www.PremierBiosoft.com), with the following criteria to identify loci with a good likelihood of reliable amplification: (i) GC content 40–60%; (ii) product size 150–350 bp; (iii) primer length 18–25 bp; (iv) melting temperature 50–60 °C with a maximum 2 °C difference between paired primers; and (v) maximum poly-N at the three prime end <3.

### 3.3. Marker Testing and Cross-species Amplification

All of the selected primer pairs were initially tested for polymorphisms in 8 individuals from the population of Longxi River. The total PCR reaction volume was 12.5 μL, containing 1.25 μL of 10X buffer, 25 mM MgCl_2_, 2.5 mM of each dNTP, 0.5 U *Taq* DNA polymerase (rTaq, TaKaRa), 3.0 pmoles each of forward and reverse primer, and ~20 ng of DNA template. The thermocycler settings for the polymerase chain reaction (PCR) were programmed as follows: 94 °C (5 min), followed by 36 cycles at 94 °C (30 s)/T_m_ (45 s)/72 °C (40 s), and a final extension at 72 °C for 7 min; the T_m_ was optimized according to different pairs of primer. The PCR products were determined based on the presence of a visible band upon running 7 μL of PCR product on a 6% denaturing polyacrylamide gel (PAGE gel). The pBR322 DNA/*MspI* molecular weight marker (TIANGEN) was used as a standard for the assessment of product size. We excluded those loci that could not be amplified or yielded double/faint bands, even after attempts to adjust the PCR conditions. Furthermore, the PCR reaction was performed twice per polymorphic locus to confirm their reproducibility.

Finally, all the successfully amplifiable microsatellite loci (including poly- and monomorphic loci) in *M. pellegrini* were assessed for cross-amplification in four other related species (*M. amblycephala*, *M. skolkoii*, *M. terminalis*, and *S. wui*), each against five individuals.

### 3.4. Statistical Analysis

The screened polymorphic loci were tested for genetic diversity at the population level, using 30 individuals collected from the Longxi River. We used POPGENE version 1.31 [42] software to determine the following summary statistics: number of alleles (N_A_), observed and expected heterozygosity (H_O_ and H_E_). Exact tests were implemented in Arlequin 3.5 [43] for the assessment of deviations from Hardy-Weinberg equilibrium (HWE) and linkage disequilibrium (LD) between the loci. CERVUS version 3.0.3 [44] was used to determine the polymorphic information content (PIC) for each locus, and the presence of null alleles was assessed at a 95% confidence interval using MICRO-CHECKER version 2.2.3 [45].

## 4. Conclusions

In this study, we developed 26 polymorphic microsatellite markers from the endangered *M. pellegrini* fish using the 454 sequencing technology. The genetic diversity of *M. pellegrini* in a wild population was moderate and could become even lower, given the continuously decreasing size of its wild population. Furthermore, the cross-species transferability of the novel markers in this study was clearly high, which might be due to the close genetic relationships among the tested species. In summary, the novel polymorphic microsatellites isolated herein will not only facilitate further parentage analyses, conservation genetic studies, and effective management of *M. pellegrini* but also be useful for exploring the genetic diversity and genetic structure of other breams in Cultrinae.

## Figures and Tables

**Table 1:** Characteristics of the 26 polymorphic microsatellite loci in *Megalobrama pellegrini* tested with 30 samples, and cross-amplification among four con-subfamilial species tested with 5 samples each (including 7 monomorphic loci screening in *M. pellegrini* shown on the last 7 rows in the table). Species abbreviations: *M. skolkoii* ‘Ms’, *M. terminalis* ‘Mt’, *M. amblycephala* ‘Ma’, and *Sinibrama wui* ‘Sw’.

Locus	Repeat motif	Primer sequence (5′–3′)		Longxi River population (N = 30)					Cross-amplification (N = 5)			

		F	R	N_A_	H_O_	H_E_	PIC	GenBank Accession No.	Ms	Mt	Ma	Sw
**MP1**	(CT)14	GCCTCACTCTATCCCTACCT	CTCAGAAACAATCCATCCAG	2	0.59	0.51	0.38	JQ087470	P	P	F	P
**MP6**	(TATC) 10	TATGATGGTGTTGCCTTGGT	GCTTTGTCCTGAGACTGTGG	4	0.15 ***	0.65	0.58	JQ087471	P	P	P	P
**MP8**	(ATCT)12	GGGGAAAATCAGAGGGAATG	GACGACGGATGGACAGACAG	3	0.50	0.57	0.46	JQ087472	P	P	P	P
**MP9**	(TTGA) 12	GTTCCGTCGTTACCAATAGAG	ACCCAAGGTCGGTCACAT	3	0.72 *	0.52	0.44	JQ087473	M	M	M	M
**MP11**	(AT)15	TGGTGAGCAGACGAAACTT	TAACCAGCGAGAACGATGT	2	0.70 **	0.46	0.35	JQ087474	M	M	M	P
**MP14**	(AATA)9	CTCGTGATGAAAGAAGAGTTAG	AATAGCCAACTGAACTGAGC	3	0.63	0.57	0.49	JQ087475	P	P	M	M
**MP15**	(TAA)10	CGTGAGATTCCCGTCTCGTC	AAAGGCAGGTGTCCCAAAAC	3	0.77	0.63	0.54	JQ087476	P	P	P	P
**MP16**	(CTAT)15	CACATTTCAGCATTTCAAGACT	TGGGTTGTTATTCTGTTTCTGA	5	0.53	0.64	0.57	JQ087477	M	P	P	P
**MP17**	(AAAC)11	TGGGGATACGGTGGAGAAC	GGTGCTGCTTGATTATTGGAG	4	0.66	0.68	0.60	JQ087478	P	F	P	F
**MP22**	(AC)16	CTGATATGAGCAAGGTAGCAA	ACTCCATTAACAATCGCACA	2	0.37	0.41	0.32	JQ087479	P	P	P	P
**MP24**	(TCAA)8	CAGACAATAGAGGGGTACACAC	TTGAATACAAGTAAGCAAAGGTT	2	0.47	0.51	0.37	JQ087480	P	P	F	F
**MP26**^#^	(TCTA)16	TGGCTGAACTCCAAAATAAG	TCACCTAAACGGGAAAATAC	4	0.27 **	0.41	0.36	JQ087481	P	P	F	F
**MP27**	(GATA)11	GAGCCACTTCACTATCGTTTA	CGCTATTGGGTTCAACATT	4	0.30	0.30	0.27	JQ087482	P	P	P	M
**MP28**	(AGAT)16	ATTCTTCCCACTGTCATTTC	CTACCCAAAACTGGCTGA	4	0.50	0.51	0.42	JQ087483	P	P	P	P
**MP29**	(TATT)11	AAAATGTCATGTCTGCTGTAT	TAAACTCTTCAAGTGGCTCA	3	0.20	0.22	0.20	JQ087484	P	P	P	P
**MP30**^#^	(GATA)11	AAGAGCCACTTCACTATCGT	GCTATTGGGTTCTAACATTG	4	0.29	0.36	0.32	JQ087485	P	P	P	P
**MP32**	(TGTT)15	GAGTCATTGAGTCCGTTTAGA	TCAGTTGAGGAGACATTTGC	4	0.77	0.65	0.56	JQ087486	P	M	M	M
**MP34**	(ATG)11	TATTCAACTTCGTGCTCCTA	GTCCATGCTTTCTGTCTTAA	2	0.77 **	0.48	0.36	JQ087487	P	P	M	M
**MP40**	(AAT)13	TATCCGTATTGCCCAAAC	TTGCTGGCATCTTACTTTC	3	0.61	0.63	0.55	JQ087488	P	P	P	M
**MP41**	(TTA)12	GGCTACAGCAGGTTTTATTT	TTACCTTTTCACCAATTCCA	2	0.14 ***	0.48	0.36	JQ087489	P	P	P	P
**MP42**	(TAA)12	TAGGAACAATGAGGGAACT	CAAGGATTAAGCCTGGTC	2	0.23	0.30	0.26	JQ087490	P	M	M	P
**MP43**	(TTA)11	GGGGACACCTTAGACTTAT	AAGTGTAAACCCTGAAGAAC	2	0.47	0.49	0.37	JQ087491	P	P	P	P
**MP45**	(AATA)8	GCGTATTTTATCATCTTTTGTGT	TGGGAGTGAAATGGAGTGAC	5	0.80	0.72	0.67	JQ087492	P	P	P	P
**MP46**	(GATA)11	GTGTGGAGTGTTTCCCTTTAGC	CCGCTATTGGGTTCAACATT	2	0.07 ***	0.50	0.37	JQ087493	P	P	P	F
**MP48**	(GGAG)15	ATGCTGTTCCAGGATCAAC	CCGCTTTTATAGCCTTTAGT	4	0.53	0.59	0.51	JQ087494	P	M	M	F
**MP55**	(TAT)12	CATACTCTGTGCATCACTTTGGTC	TTTACGAGGGCTTATTAGGGC	2	0.30	0.48	0.36	JQ087495	P	P	P	P
**MP2**	(CA)18	TGTCCTCATAAGTCACCCTC	ACACCGTCTAATCTGCCTAC	1	0.00	0.00	M		M	M	M	P
**MP3**	(AC)21	ACAACACTTCACCACCCA	TAAGAACTACAAATACCCACTG	1	0.00	0.00	M		F	F	F	F
**MP5**	(AGAT)9	AGTCCTCTGCCACCTCCTG	GCTACTTGACCCTTCATCATACA	1	0.00	0.00	M		P	P	P	P
**MP19**	(TAT) 10	AAAGGGATAGCAGACAAGATAC	ACAACCCAAACAGGTGAAT	1	0.00	0.00	M		M	P	F	F
**MP23**	(AGA)11	AGTTTTCGAGCACGTTTAGG	CCCACTGTTCACTGTTTTCC	1	0.00	0.00	M		M	M	M	M
**MP25**	(AAAC)14	AACAGGGGAGGGGAAGATTA	CTATTGAAGAGGCAAAGCGAG	1	0.00	0.00	M		P	P	P	P
**MP50**	(TTTC)14	CGTAATGTTTGGTGTCGTTTTG	AATGTGGGCGGGGTTTAGT	1	0.00	0.00	M		M	M	M	M

‘#’ locus in linkage disequilibrium with each other, ‘N_A_’ the number of alleles, ‘H_O_’ observed heterozygosity, ‘H_E_’ expected heterozygosity, and significance of deviation from Hardy-Weinberg equilibrium at P-levels 0.05(*), 0.01(**), 0.001(***),‘PIC’ the polymorphism information content, ‘P’ polymorphic, ‘M’ monomorphic, ‘F’ failed to amplify or multiple non-specific amplification.

## References

[1] Luo Y.L. (1990). A revision of fishes of the cyprinid genus *Megalobrama*. Acta Hydrobiol. Sin.

[2] Chen Y.Y. (1998). Fauna Sinica, Osteichthyes Cypriniformes II (in Chinese).

[3] Park Y.S., Chang J.B., Lek S., Cao W.X., Brosse S. (2003). Conservation strategies for endemic fish species threatened by the Three Gorges Dam. Conserv. Biol.

[4] Liu J. (2004). A quantitative analysis on threat and priority of conservation order of the endemic fishes in upper reaches of the Yangtze River. China Environ. Sci.

[5] Li W.J., Wang J.W., Xie C.X., Tan D.Q. (2007). Reproductive biology and spawning habitats of *Megalobrama pellegrini*, an endemic fish in upper-reaches of Yangtze River basin. Acta Ecol. Sin.

[6] Gao X., Tan D.Q., Liu H.Z., Wang J.W. (2009). Exploitation status and conservation of a population of *Megalobrama pellegrini* in Longxi river in the upper Yangtze River basin. Sichuan J. Zool.

[7] Wang J.W., Tan D.Q., Li W.J. (2005). Preliminary studies on artificial propagation and embryonic development of *Megalobrama pellegrini*. Acta Hydrobiol. Sin.

[8] Li W.J., Wang J.W., Tan D.Q., Dan S.G. (2005). Observation on postembryonic development of *Megalobrama pellegrini*. J. Fish. China.

[9] Gao X., Liu H.Z., Wang J.W. (2008). Applicaiton of logistic regression analysis on study of life history pattern of *Megalobrama pellegrini*. Sichuan. J. Zool.

[10] Zhu Z.Q., Li Y., Zheng K.D., Zhu X.Z., Liu B., Zhang L. (2009). Cloning and sequence analysis of encoding cDNA sequence of Neuropeptide Y in *Megalobrama pellegrini*. Freshw. Fish.

[11] Liu H.Z., Wang Y.P. (1997). Studies on genetic structure and null allele in a natural population of *Megalobrama pellegrini*. Acta Hydrobiol. Sin.

[12] Xu W., Xiong B.X. (2008). Advances in the research on genus *Megalobrama* in China. J. Hydroecol.

[13] Cai M.J., Zhang M.Y., Zeng Q.L., Liu H.Z. (2001). A study on the morphological of the genus *Megalobrama*. Acta Hydrobiol. Sin.

[14] Mittal N., Dubey A. (2009). Microsatellite markers-a new practice of DNA based markers in molecular genetics. Phcog. Rev.

[15] Jones A.G., Small C.M., Paczolt K.A., Ratterman N.L. (2010). A practical guide to methods of parentage analysis. Mol. Ecol. Resour.

[16] Goldstein D.B., Schlotterer C (1999). Microsatellites, Evolution and Applications.

[17] Ellegren H. (2004). Microsatellites, simple sequences with complex evolution. Nat. Rev. Genet.

[18] Glenn T.C., Schable N.A., Zimmer E.A., Roalson E.H. (2005). Isolating Microsatellite DNA Loci. Methods in Enzymology, Molecular Evolution, Producing the Biochemical Data, Part B.

[19] Abdelkrim J., Robertson B.C., Stanton J.-A.L., Gemmell N.J. (2009). Fast, cost-effective development of species-specific microsatellite markers by genomic sequencing. BioTechniques.

[20] Allentoft M.E., Schuster S.C., Holdaway R.N., Hale M.L., McLay E., Oskam C., Gilbert T.P., Spencer P., Willerslev E., Bunce M. (2009). Identification of microsatellites from an extinct moa species using high-throughput (454) sequence data. BioTechniques.

[21] Santana Q.C., Coetzee M.P.A., Steenkamp E.T., Mlonyeni O.X., Hammond G.N.A., Wingfield M.J., Wingfield B.D. (2009). Microsatellite discovery by deep sequencing of enriched genomic libraries. BioTechniques.

[22] Castoe T.A., Poole A.W., Gu W.J., Jason de Koning A.P., Daza J.M., Smith E.N., Pollock D.D. (2009). Rapid identification of thousands of copperhead snake (*Agkistrodon contortrix*) microsatellite loci from modest amounts of 454 shotgun genome sequence. Mol. Ecol. Resour.

[23] Csencsics D., Brodbeck S., Holderegger R. (2010). Cost-effective, species-specific microsatellite development for the endangered dwarf bulrush (*Typha minima*) uing next-generation sequencing technology. J. Hered.

[24] Saarinen E.V., Austin J.D. (2010). When technology meets conservation, increased microsatellite marker production using 454 genome sequencing on the endangered okaloosa darter (*Etheostoma okaloosae*). J. Hered.

[25] Gardner M.G., Fitch A.J., Bertozzi T., Lowe A.J. (2011). Rise of the machines—Recommendations for ecologists when using next generation sequencing for microsatellite development. Mol. Ecol. Resour.

[26] McCulloch E.S., Stevens R.D. (2011). Rapid development and screening of microsatellite loci for *Artibeus lituratus* and their utility for six related species within Phyllostomidae. Mol. Ecol. Resour.

[27] Perry J.C., Rowe L. (2011). Rapid microsatellite development for water striders by next-generation sequencing. J. Hered.

[28] Wood R., Weyeneth N., Appleton B. (2011). Development and characterisation of 20 microsatellite loci isolated from the large bent-wing bat, *Miniopterus schreibersii* (Chiroptera, Miniopteridae) and their cross-taxa utility in the family Miniopteridae. Mol. Ecol. Resour.

[29] Guichoux E., Lagache L., Wagner S., Chaumeil P., Leger P., Lepais O., Lepoittevin C., Malausa T., Revardel E., Salin F., Petit R.J. (2011). Current trends in microsatellite genotyping. Mol. Ecol. Resour.

[30] Li W.T., Liao X.L., Yu X.M., Wang D., Tong J.G. (2007). Isolation and characterization of polymorphic microsatellite loci in Wuchang bream (*Megalobrama amblycephala*). Mol. Ecol. Notes.

[31] Lai Y., Sun F. (2003). The relationship between microsatellite slippage mutation rate and the number of repeat units. Mol. Biol. Evol.

[32] Botstein D., White R.L., Skolnick M., Davis R.W. (1980). Construction of a genetic linkage map in man using restriction fragment length polymorphisms. Am. J. Hum. Genet.

[33] Aliah R.S., Takagi M., Dong S., Teoh C.T., Taniguchi N. (1999). Isolation and inheritance of microsatelllite markers in the common carp *Cyprinus carpio*. Fish Sci.

[34] Du C.B., Sun X.W., Lou Y.D., Shen J.B. (2000). The genetic heterozygosity analysis to wild carp and two cultivated strains of common carp using microsatellite technique. J. Shanghai Fish Univ.

[35] Wang W., You F., Gao T.X., Zhang P.J. (2004). Genetic variations at ten microsatellite loci in natural and cultured stocks of left-eyed flounder *Paralichthys olivaceu* in Shandong coastal waters. Oceanol. Limnol. Sin.

[36] Sumantadinata K., Taniguchi N. (1990). Comparison of electrophoretic allele frequencies and genetic variability of common carp stocks from Indonesia and Japan. Aquaculture.

[37] Liao X.L., Yu X.M., Chang J.B., Tong J.G. (2007). Polymorphic microsatellites in largemouth bronze gudgeon (*Coreius guichenoti*) developed from repeat-enriched libraries and cross-species amplifications. Mol. Ecol. Notes.

[38] Liao X.L., Wang D., Yu X.M., Li W.T., Cheng L., Wang J.W., Tong J.G. (2007). Characterization of novel microsatellite loci in rare minnow (*Gobiocypris rarus*) and amplification in closely related species in Gobioninae. Conserv. Genet.

[39] Yue H., Yuan H., Zhang X.Y. (2009). Fifteen novel polymorphic microsatellites in rock carp, *Procypris rabaudi* (Tchang), an endemic fish species in the upper reaches of the Yangtze River drainage. Conserv. Genet.

[40] Zhu D., Chang J.B. (2008). Annual variations of biotic integrity in the upper Yangtze River using an adapted index of biotic integrity (IBI). Ecol. Indic.

[41] Faircloth B.C. (2008). Msatcommander, detection of microsatellite repeat arrays and automated, locus-specific primer design. Mol. Ecol. Resour.

[42] Yeh F.C., Yang R.C., Boyle T (1999). POPGENE Microsoft Windows-based Freeware for Population Genetic Analysis Release 1.31.

[43] Excoffier L., Laval G., Schneider S. (2005). Arlequin ver. 3.0: An integrated software package for population genetics data analysis. Evol. Bioinforma. Online.

[44] Kalinowski S., Taper M., Marshall T. (2007). Revising how the computer program CERVUS accommodates genotyping error increases success in paternity assignment. Mol. Ecol. Notes.

[45] Van Oosterhout C., Hutchinson W.F., Wills D.P.M., Shipley P. (2004). MICRO-CHECKER, software for identifying and correcting genotyping errors in microsatellite data. Mol. Ecol. Notes.

